# Angiographic and early in-hospital profile of primary percutaneous coronary intervention in patients with substance use disorder presenting with ST-segment elevation myocardial infarction

**DOI:** 10.1038/s41598-026-65981-9

**Published:** 2026-08-15

**Authors:** Khaled M. Elmaghraby, Abdalla A. Gamal, Hatem Abdel Rahman Helmy, Yousra Ghzally

**Affiliations:** Department of Cardiovascular Medicine, Faculty of Medicine, Assiut University, Assiut, 71515 Egypt

**Keywords:** Substance use disorder, ST-segment elevation myocardial infarction, Primary percutaneous coronary intervention, Glycoprotein IIb/IIIa inhibitors, Thrombus-rich STEMI phenotype, Cardiology, Medical research

## Abstract

Acute myocardial infarction is increasingly recognized in younger adults, but the angiographic and early clinical profile of ST-segment elevation myocardial infarction among patients with substance use disorder remains incompletely defined. This single-center prospective observational cohort study evaluated the angiographic and early in-hospital profile after primary percutaneous coronary intervention in patients with and without substance use disorder. A total of 175 patients formed the final analytic cohort, including 75 patients with substance use disorder confirmed by DSM-5 criteria supported by point-of-care urine toxicology screening and 100 patients without substance use disorder. Patients with substance use disorder were younger, exclusively male, and had fewer traditional cardiometabolic co-morbidities. In unadjusted analyses, they more often had single-vessel coronary disease and more frequent glycoprotein IIb/IIIa inhibitor use, whereas angiographic thrombus presence was similarly frequent in both groups. After multivariable adjustment, the associations with single-vessel disease and glycoprotein IIb/IIIa inhibitor use were not statistically robust. Patients with substance use disorder presented with a distinct baseline and procedural profile, but adjusted analyses did not support an independent association with the principal angiographic or procedural outcomes assessed, supporting careful toxicology assessment and thrombus-focused procedural planning while avoiding causal interpretation.

## Introduction

The incidence of ST-segment elevation myocardial infarction (STEMI) among young adults is clinically important because the risk-factor profile, mechanism of coronary occlusion, angiographic pattern, and short-term prognosis may differ from those in older patients. The proportion of acute myocardial infarction occurring among very young adults has been estimated at approximately 2 to 6%. Contemporary contributors include tobacco exposure, substance use, metabolic risk, vasomotor dysfunction, endothelial injury, platelet activation, and other factors that may interact with plaque erosion, plaque rupture, and thrombosis^1^.

In Egypt, acute coronary syndrome affects a substantial proportion of young and middle-aged adults. Bashandy et al. reported that among 184 patients admitted with acute coronary syndrome, 66.3% were either young or middle-aged, and 52.2% presented with STEMI^2^. Previous studies have shown that younger patients with STEMI are predominantly male, less often have diabetes or hypertension, and more often have single-vessel coronary disease than older patients^3–5^.

Substance use disorders are prevalent in Middle Eastern Arab countries, including Egypt, yet data remain limited on patients with substance use disorder who present to emergency departments and catheterization laboratories with acute coronary syndromes^6^. Current chest-pain guidance recommends considering substance exposure in selected patients, particularly younger patients or those with clinical suspicion of substance use^7^. Cannabis, tramadol, opioids, cocaine, and amphetamine exposure have each been linked to coronary vasospasm, endothelial dysfunction, platelet activation, a prothrombotic milieu, arrhythmia risk, and premature myocardial infarction^8–13^.

The contemporary clinical evidence is heterogeneous. In a 2023 case–control study of 186 patients with STEMI undergoing emergency PCI, Amirpour et al. found that opioid-dependent patients were younger and more often smoked, but post-PCI TIMI grade 3 flow, in-hospital complications, and mortality did not differ from those of patients without opioid dependence^14^. In contrast, the 2024 multicenter French ADDICT-ICCU study used systematic urine multidrug testing in 325 patients with STEMI and detected recent recreational drug exposure in 12.6% overall and 34.0% of patients younger than 50 years. Exposed patients were younger, predominantly male, and more often current smokers, and they experienced more in-hospital major adverse cardiac events and ventricular arrhythmias after multivariable adjustment^15^. These divergent findings indicate that substance type, exposure definition, cohort composition, and outcome ascertainment materially influence the observed associations.

Broader recent evidence also suggests potential longer-term cardiovascular risk. A Canadian population-based cohort associated cannabis use disorder with a higher incidence of adverse cardiovascular events^16^, while a 10-year follow-up study of patients undergoing PCI associated opium consumption with increased all-cause mortality and major adverse cardiac and cerebrovascular events^17^. Systematic reviews published in 2024 and 2025 reported associations between cannabis exposure and myocardial infarction or other major cardiovascular outcomes, but emphasized residual confounding, tobacco co-use, variable exposure definitions, and the observational nature of the evidence^18,19^.

Despite this growing literature, important gaps remain. Most studies are retrospective, administrative, substance-specific, or dominated by cocaine, amphetamine, or cannabis exposure. Few combine DSM-based clinical assessment with systematic toxicology screening, direct angiographic characterization, and prospectively verified early outcomes in patients undergoing primary PCI. Evidence from North Africa and from cohorts in which cannabis, tramadol, and opium are the dominant exposures is particularly limited. This study addresses that gap by comparing the baseline clinical phenotype, angiographic findings, procedural treatment patterns, and available early in-hospital outcomes of patients with and without substance use disorder who presented with STEMI and underwent primary PCI.

The aim was to evaluate the angiographic and early in-hospital profile of patients undergoing primary PCI for STEMI, comparing patients with substance use disorder with patients without substance use disorder.

## Methods

This was a single-center prospective observational cohort study conducted at cardiovascular medicine department, Assiut University Heart Hospital, Assiut, Egypt, over 6 months from December 1, 2022, to May 31, 2023. The study was approved by the Institutional Review Board of the Faculty of Medicine, Assiut University (approval no. 17101052), and complied with the Declaration of Helsinki. Confidentiality and privacy of all data were assured. Written informed consent was obtained from each participant after the study aim was explained. The research project was registered at ClinicalTrials.gov (NCT04090216).

Study population. Consecutive adults aged 18 years or older presenting with STEMI and eligible for primary PCI at Assiut University Heart Hospital were screened. Patients were excluded if they presented with STEMI but did not undergo primary PCI, died within 6 h of presentation before an accurate exposure history and urine sample could be obtained, had previous STEMI, or had prior revascularization by PCI or coronary artery bypass grafting. The early-death exclusion was required for exposure ascertainment, but it creates survivor-selection bias by removing the sickest patients. It may lower the observed frequency of shock, malignant arrhythmia, and mortality, and may distort between-group associations if substance use disorder was differentially represented among the 31 early deaths. Because reliable exposure status was unavailable for these patients, the direction and magnitude of this bias cannot be quantified.

STEMI was diagnosed using compatible ischemic symptoms, persistent ST-segment elevation in at least 2 contiguous leads on 12-lead electrocardiography, and cardiac biomarker elevation, in accordance with the Fourth Universal Definition of Myocardial Infarction and contemporary STEMI guidance^20,21^.

Exposure definition and substance use disorder assessment. Patients were classified into 2 groups. The substance use disorder group included patients who fulfilled DSM-5 criteria for substance use disorder and had supportive point-of-care urine toxicology screening. The non-substance use disorder group included patients without clinical evidence of substance use disorder and without supportive urine-screen evidence. DSM-5 criteria were applied by study physicians using clinical history and admission interview data after clinical stabilization^34^.

Urine toxicology screening. Emergency urine samples were collected from all included patients after admission, before primary PCI when clinically feasible, or within 18 h after primary PCI. Urine drug screening was performed using the Wondfo One Step Multi-Drug Urine Test Panel (Guangzhou Wondfo Biotech Co., Ltd., China), a rapid qualitative point-of-care competitive immunoassay. The panel screened for six substance classes: tetrahydrocannabinol, tramadol, opiates/morphine, cocaine, amphetamine, and benzodiazepines. Results were interpreted visually within the manufacturer-specified reading window using manufacturer-defined positivity thresholds. The assay was used only as a screening test and positive results were not confirmed by gas chromatography–mass spectrometry or liquid chromatography-mass spectrometry. Accordingly, the assay could not quantify exposure, distinguish prescribed or therapeutic use from nonmedical use, identify substances outside the panel, or eliminate class cross-reactivity, variable detection windows, sample dilution, and false-positive or false-negative results. Urine toxicology therefore supported, but did not independently establish, the exposure classification.

Cardiovascular risk factors, co-morbidities, substance type, chest-pain duration, presenting symptoms, admission vital signs, physical examination findings, electrocardiographic findings, routine laboratory results, and echocardiographic findings were prospectively recorded. Diabetes was defined as a prior diagnosis, treatment for diabetes, fasting plasma glucose greater than 126 mg/dL, or hemoglobin A1c greater than 6.5%. Hypertension was defined as a prior diagnosis or treatment for hypertension, or systolic blood pressure 140 mm Hg or higher, or diastolic blood pressure 90 mm Hg or higher. Smoking was classified as current, former, or never; current smoking was defined as tobacco use within the preceding month.

Rationale for selected patient characteristics. Baseline characteristics were prespecified because they are clinically related to premature coronary disease, angiographic disease burden, procedural treatment, or early STEMI prognosis and could confound comparisons by substance use disorder status. Age, sex, and current smoking characterize demographic and exposure patterns; diabetes, hypertension, chronic kidney disease, and liver disease characterize chronic cardiometabolic, renal, and bleeding-risk burden; admission systolic blood pressure and chest-pain duration characterize acute hemodynamic status and ischemic time. These variables were collected using uniform definitions and were not selected solely according to univariable P values. Their principal purpose was to describe case-mix, guide cautious adjustment, and make the limits of between-group comparability explicit, rather than to imply a causal pathway.

Echocardiographic assessment included left ventricular dimensions and volumes, systolic function, diastolic function, segmental wall-motion abnormalities, mitral regurgitation, and mechanical complications when available.

The number of diseased vessels, the culprit vessel, the presence of angiographic thrombus, and available procedural variables were recorded. Glycoprotein IIb/IIIa inhibitor use was recorded as an adjunctive procedural treatment and was used only as an operational treatment-triggered marker of operator-determined difficult thrombus management. It was not treated as a validated angiographic thrombus burden score and cannot distinguish plaque rupture, plaque erosion, vasospasm with secondary thrombosis, embolic phenomena, or unmeasured thrombophilia.

Available in-hospital assessment focused on verified events and markers, including rhythm-derived arrhythmia or conduction abnormality and renal injury markers. Rare clinical events were interpreted cautiously and were not used for unsupported comparative inference when source data verification was insufficient.

Killip classification. Killip class I indicated no clinical signs of heart failure; class II indicated rales or crackles, S3, or elevated jugular venous pressure; class III indicated acute pulmonary edema; and class IV indicated cardiogenic shock or hypotension with evidence of peripheral hypoperfusion^22^.

## Statistical analysis

Data entry and initial descriptive analysis were performed using SPSS version 22 (IBM Corp., Armonk, NY, USA). Categorical variables were summarized as counts and percentages, with percentages calculated after excluding missing observations from the denominator. Continuous variables were assessed for distributional form and summarized as mean (SD) when approximately normally distributed or median (interquartile range) when skewed. Between-group comparisons used the Pearson chi-square test or Fisher’s exact test for categorical variables, the independent-samples t test for approximately normally distributed continuous variables, and the Mann–Whitney U test for skewed continuous variables. Exact P values are reported when available, with P < 0.001 reserved for values that are very small.

Because this was an exploratory, single-center, observational cohort study of consecutive eligible STEMI presentations during a predefined 6-month recruitment interval, no formal a priori sample size calculation was performed. The final sample size was determined by the number of eligible patients during the recruitment period. Analyses of uncommon in-hospital events were therefore considered underpowered and hypothesis-generating.

The exposure groups differed substantially at baseline, including age, sex, smoking, diabetes, hypertension, chronic kidney disease, liver disease, and admission hemodynamics. All comparative findings were therefore interpreted as associations rather than causal effects. Exploratory multivariable regression models were fitted for the main patient-level outcomes in the dataset. Binary angiographic and procedural outcomes were analyzed using logistic regression, while continuous echocardiographic outcomes were analyzed using linear regression with heteroskedasticity-consistent standard errors. Models included substance use disorder status, age, sex, current smoking, diabetes mellitus, hypertension, chronic kidney disease, admission systolic blood pressure, and chest-pain duration. Covariates were prespecified according to clinical relevance rather than selected by stepwise procedures or univariable statistical significance. Adjusted estimates are reported as odds ratios for binary outcomes and beta coefficients for continuous outcomes, with 95% confidence intervals.

Model stability and validation. The single-vessel disease model included 88 outcome events and the glycoprotein IIb/IIIa inhibitor model included 57 events across 9 predictor terms, corresponding to approximately 9.8 and 6.3 events per predictor, respectively. The latter is particularly vulnerable to overfitting, and complete or near-complete separation for sex, current smoking, and chronic kidney disease further reduced effective information. No interaction testing, data-driven predictor selection, bootstrap optimism correction, cross-validation, or external validation was performed. Because the models were exploratory explanatory models rather than clinical prediction tools, discrimination and calibration indices were not used to claim predictive performance. Wide confidence intervals, coefficient instability, and the non-estimable thrombus model were treated as indicators of limited model reliability. Reporting followed the STROBE framework^23^.

## Results

**Fig. 1:**
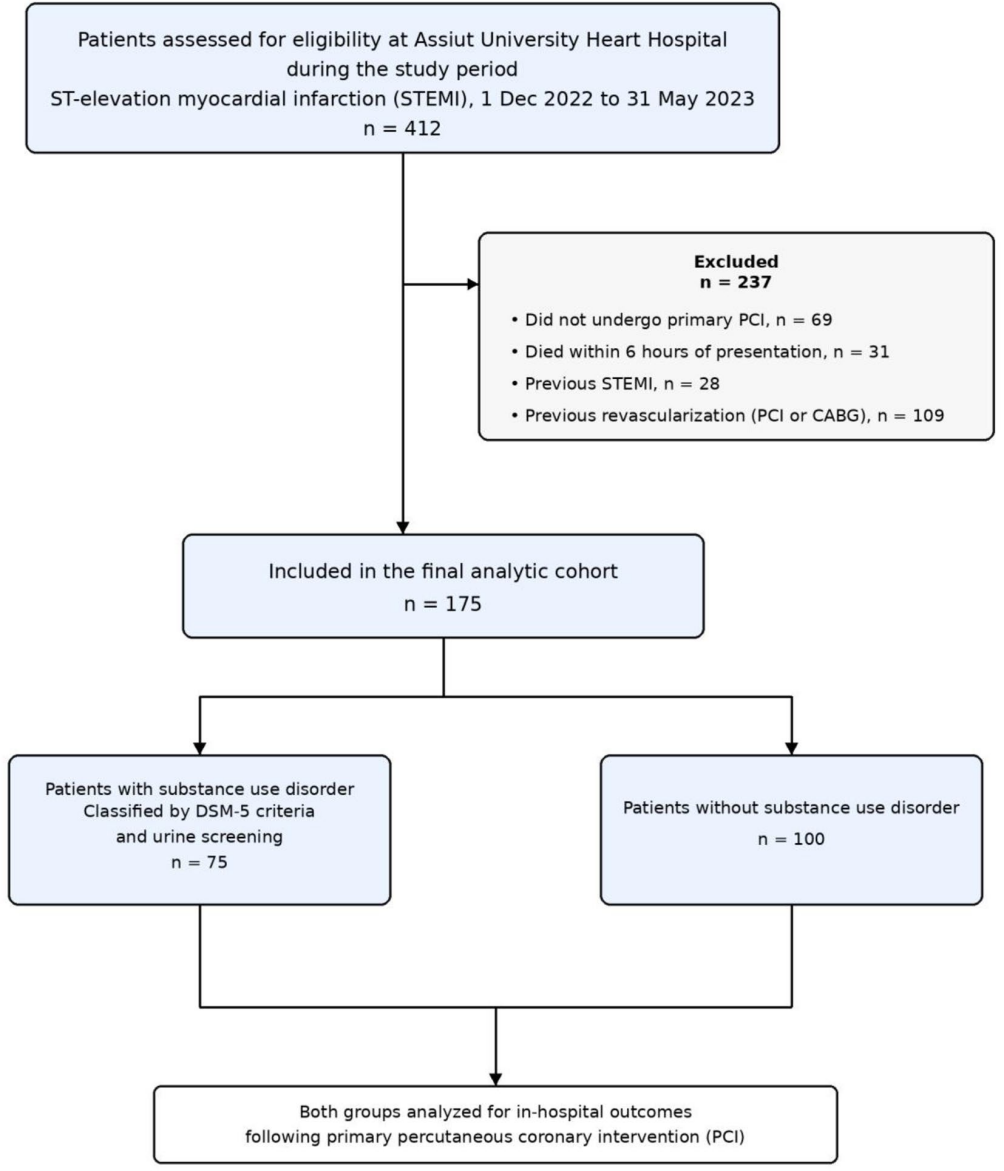
Patient-flow diagram of the study cohort. Consecutive patients with ST-segment elevation myocardial infarction were screened, exclusions were applied, and the final analytic cohort was classified into patients with and without substance use disorder.

During the study period, 412 patients with STEMI were assessed for eligibility. A total of 237 patients were excluded: 69 did not undergo primary PCI, 31 died within 6 h of presentation, 28 had previous STEMI, and 109 had previous revascularization by PCI or coronary artery bypass grafting. The final analytic cohort included 175 patients: 75 patients with substance use disorder (42.9%) and 100 patients without substance use disorder (57.1%) (Fig. 1).

Baseline phenotype. Patients with substance use disorder were substantially younger than patients without substance use disorder (43.2 [8.9] vs 63.8 [6.8] years). All patients in the substance use disorder group were male, whereas women accounted for 36.0% of the non-substance use disorder group. Traditional cardiometabolic co-morbidities, including diabetes, hypertension, chronic kidney disease, and liver disease, were more prevalent among patients without substance use disorder, whereas all patients with substance use disorder were current smokers. These imbalances are central to interpretation and indicate substantial confounding by age, sex, tobacco exposure, and co-morbidity burden (Table 1).

**Table 1:** Baseline, clinical, and laboratory characteristics.

Characteristic	Patients with substance use disorder (n = 75)	Patients without substance use disorder (n = 100)	P value
Age, years, mean (SD)	43.2 (8.9)	63.8 (6.8)	P < 0.001
Male sex, n/N (%)	75/75 (100.0)	64/100 (64.0)	P < 0.001
Current smoking, n/N (%)	75/75 (100.0)	54/100 (54.0)	P < 0.001
Diabetes mellitus, n/N (%)	5/75 (6.7)	48/100 (48.0)	P < 0.001
Hypertension, n/N (%)	1/75 (1.3)	65/100 (65.0)	P < 0.001
Prior cerebrovascular stroke, n/N (%)	1/75 (1.3)	1/100 (1.0)	P = 1.000
Chronic kidney disease, n/N (%)	0/75 (0.0)	6/100 (6.0)	P = 0.038
Liver disease, n/N (%)	1/75 (1.3)	7/100 (7.0)	P = 0.140
Chest pain duration, hours, mean (SD)	5.4 (3.8)	7.5 (5.2)	P = 0.003
Pulse, beats/min, mean (SD)	82.5 (16.2)	84.3 (14.1)	P = 0.422
Systolic blood pressure, mm Hg, mean (SD)	116.3 (17.3)	130.2 (13.9)	P < 0.001
Diastolic blood pressure, mm Hg, mean (SD)	72.0 (10.2)	80.2 (8.0)	P < 0.001
Hemoglobin, g/dL, mean (SD)	14.2 (1.5)	13.4 (1.6)	P = 0.001
Dyspnea, n/N (%)	5/75 (6.7)	29/100 (29.0)	P < 0.001
Vomiting, n/N (%)	25/75 (33.3)	13/100 (13.0)	P = 0.001
White blood cells, 10^3/uL, mean (SD)	12.6 (3.9)	10.4 (3.2)	P < 0.001
Platelets, 10^3/uL, mean (SD)	288.9 (95.2)	294.1 (74.6)	P = 0.697
Urea, mg/dL, mean (SD)	5.7 (4.9)	8.0 (4.2)	P = 0.001
Creatinine, mg/dL, mean (SD)	0.75 (0.16)	0.93 (0.46)	P < 0.001
Troponin, median (IQR)	33.0 (18.9 to 42.5)	41.5 (23.9 to 71.8)	P = 0.002
Electrolyte abnormality, n/N (%)	8/75 (10.7)	12/100 (12.0)	P = 0.784

Values are n/N (%) unless otherwise stated. P values were generated using independent samples t tests for continuous approximately normally distributed variables, Mann–Whitney U test for troponin, and chi-square or Fisher exact tests for categorical variables, as appropriate.

**Fig. 2:**
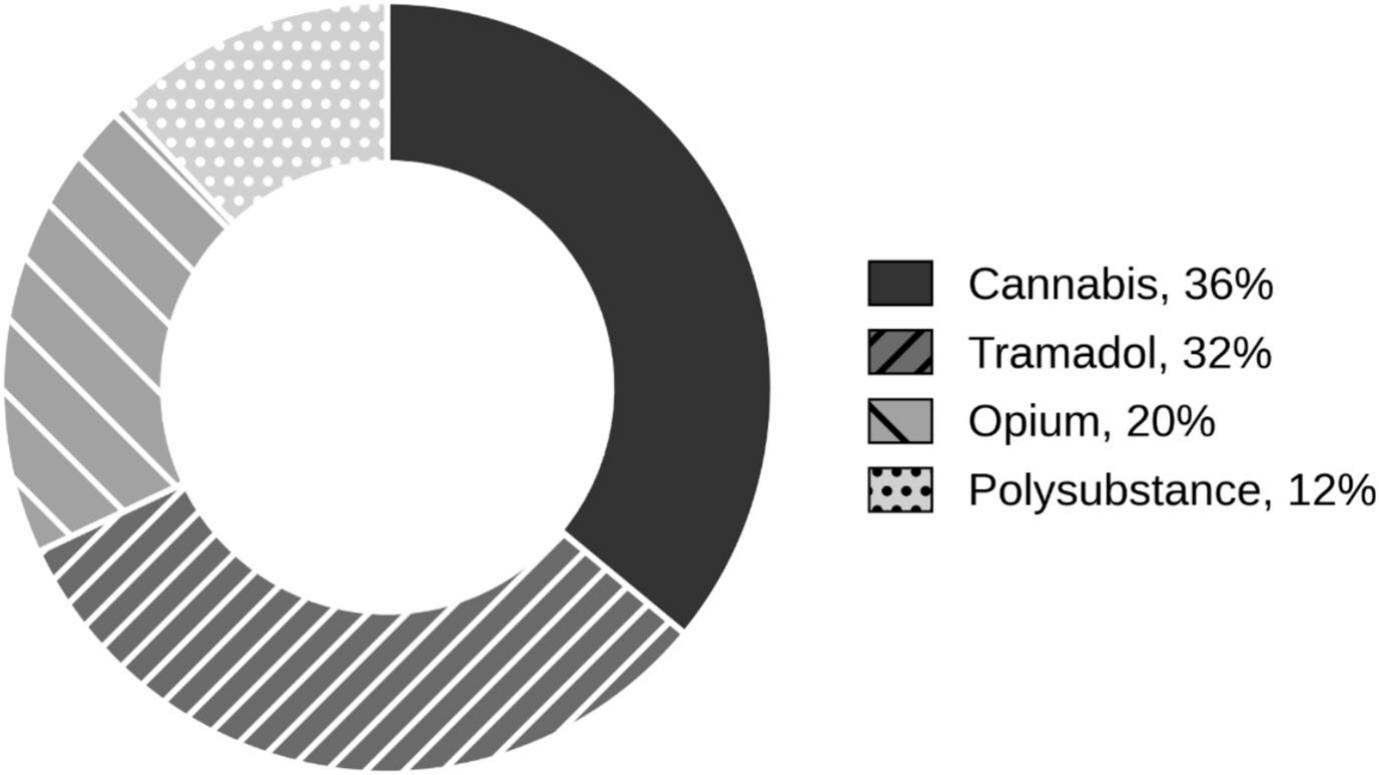
Distribution of reported substance categories among patients with substance use disorder.

Substance profile. Among patients with substance use disorder, 27 (36.0%) used cannabis, 24 (32.0%) used tramadol, 15 (20.0%) used opium, and 9 (12.0%) used multiple substances. This local exposure pattern differs from cohorts dominated by cocaine or amphetamine exposure (Fig. 2)

Clinical presentation and laboratory findings. Chest pain and dyspnea were the main presenting symptoms. Chest-pain duration was shorter among patients with substance use disorder than among patients without substance use disorder (5.4 [3.8] vs 7.5 [5.2] hours). Admission systolic and diastolic blood pressures were lower among patients with substance use disorder. White blood cell count and hemoglobin were higher among patients with substance use disorder, whereas urea, creatinine, and troponin values were generally higher or more abnormal among patients without substance use disorder (Table 1).

Electrocardiographic and echocardiographic findings. Anterior STEMI was the most common infarct territory in both groups. Left ventricular ejection fraction and the number of affected cardiac segments did not differ meaningfully between groups. Diastolic function was dominated by grade 1 impaired relaxation in both groups. Mitral regurgitation were more frequent among patients without substance use disorder, consistent with their older age and greater co-morbidity burden (Table 2).

**Table 2:** Electrocardiographic and echocardiographic findings.

Finding	Patients with substance use disorder (n = 75)	Patients without substance use disorder (n = 100)	P value
Anterior STEMI territory, n/N (%)	46/75 (61.3)	64/100 (64.0)	P = 0.718
Inferior STEMI territory, n/N (%)	27/75 (36.0)	34/100 (34.0)	P = 0.783
Lateral STEMI territory, n/N (%)	2/75 (2.7)	4/100 (4.0)	P = 0.702
Posterior STEMI territory, n/N (%)	4/75 (5.3)	1/100 (1.0)	P = 0.166
Right ventricular STEMI territory, n/N (%)	5/75 (6.7)	1/100 (1.0)	P = 0.085
Any rhythm or conduction abnormality, n/N (%)	6/75 (8.0)	5/100 (5.0)	P = 0.533
Ventricular tachycardia/fibrillation, n/N (%)	4/75 (5.3)	0/100 (0.0)	P = 0.032
Atrial fibrillation, n/N (%)	1/75 (1.3)	2/100 (2.0)	P = 1.000
Heart block, n/N (%)	0/75 (0.0)	2/100 (2.0)	P = 0.507
Left ventricular ejection fraction, %, mean (SD)	49.4 (7.0)	48.1 (7.7)	P = 0.248
Affected cardiac segments, median (IQR)	5.0 (3.2 to 6.0)	5.0 (4.0 to 6.8)	P = 0.694
Diastolic function: normal	2/75 (2.7)	0/100 (0.0)	P = 0.090
Diastolic function: impaired relaxation, grade 1	69/75 (92.0)	88/100 (88.0)	
Diastolic function: grade 2	4/75 (5.3)	12/100 (12.0)	
Mitral regurgitation: none	65/75 (86.7)	68/100 (68.0)	P = 0.054
Mitral regurgitation: trace	1/75 (1.3)	4/100 (4.0)	
Mitral regurgitation: mild	7/75 (9.3)	19/100 (19.0)	
Mitral regurgitation: moderate	2/75 (2.7)	5/100 (5.0)	
Mitral regurgitation: severe	0/75 (0.0)	4/100 (4.0)	

Values are n/N (%) unless otherwise stated. STEMI territory variables are not mutually exclusive when multiterritory involvement was recorded. The P value for multilevel variables is shown once for the whole distribution.

Angiographic and procedural findings. Patients with substance use disorder more often had single-vessel disease, whereas patients without substance use disorder more often had multivessel coronary disease. The left anterior descending artery was the most frequent culprit vessel in both groups. Angiographic thrombus was frequent in both groups, affecting 72 of 75 patients with substance use disorder (96.0%) and 96 of 100 patients without substance use disorder (96.0%), so thrombus presence itself did not distinguish the cohorts (Table 3).

**Table 3:** Angiographic and procedural findings.

Finding	Patients with substance use disorder (n = 75)	Patients without substance use disorder (n = 100)	P value
Single-vessel disease	50/75 (66.7)	38/100 (38.0)	P < 0.001
Two-vessel disease	17/75 (22.7)	27/100 (27.0)	
Three or more diseased vessels	8/75 (10.7)	35/100 (35.0)	
Culprit vessel: LAD	47/75 (62.7)	63/100 (63.0)	P = 0.663
Culprit vessel: RCA	24/75 (32.0)	28/100 (28.0)	
Culprit vessel: LCX	4/75 (5.3)	5/100 (5.0)	
Culprit vessel: OM	0/75 (0.0)	2/100 (2.0)	
Culprit vessel: diagonal	0/75 (0.0)	1/100 (1.0)	
Culprit vessel: left main	0/75 (0.0)	1/100 (1.0)	
Angiographic thrombus present	72/75 (96.0)	96/100 (96.0)	P = 1.000
Glycoprotein IIb/IIIa inhibitor use	33/75 (44.0)	24/100 (24.0)	P = 0.005
Angiographic calcification	0/75 (0.0)	10/100 (10.0)	P = 0.005

Values are n/N (%) unless otherwise stated. Culprit-vessel P value refers to the overall distribution across available culprit-vessel categories. LAD, left anterior descending artery; LCX, left circumflex artery; RCA, right coronary artery; OM, obtuse marginal branch; LM, left main coronary artery.

In-hospital outcome interpretation. Available in-hospital outcome data did not show a statistically robust between-group difference in Killip class distribution, gastrointestinal bleeding, in-stent thrombosis, recurrent chest pain, acute kidney injury, or in-hospital mortality. These events were uncommon and were not suitable for stable adjusted modeling. They should therefore be interpreted as descriptive, hypothesis-generating findings rather than evidence of equivalence between groups (Table 4).

**Table 4:** Early in-hospital outcomes.

Outcome	Patients with substance use disorder (n = 75)	Patients without substance use disorder (n = 100)	P value
Killip class I	61 (81.3%)	77 (77.0%)	P = 0.740
Killip class II-III	11 (14.7%)	17 (17.0%)	P = 0.740
Killip class IV	3 (4.0%)	6 (6.0%)	P = 0.740
Ventricular tachycardia	7 (9.3%)	1 (1.0%)	P = 0.163
Atrial fibrillation	2 (2.6%)	2 (2.0%)	P = 0.163
Heart block	4 (5.3%)	5 (5.0%)	P = 0.163
Gastrointestinal bleeding	1 (1.3%)	6 (6.0%)	P = 0.110
In-stent thrombosis or recurrent chest pain	4 (5.3%)	3 (3.0%)	P = 0.464
Acute kidney injury	0 (0.0%)	4 (4.0%)	P = 0.550
In-hospital mortality	0 (0.0%)	0 (0.0%)	Not estimable

Glycoprotein IIb/IIIa inhibitors were used more frequently in patients with substance use disorder than in patients without substance use disorder in unadjusted analysis (33 of 75 [44.0%] vs 24 of 100 [24.0%]). However, after adjustment for age, sex, smoking, diabetes mellitus, hypertension, chronic kidney disease, admission systolic blood pressure, and chest pain duration, the association was not statistically significant (adjusted odds ratio, 0.31; 95% CI, 0.09 to 1.06; P = 0.062). Similarly, the unadjusted association with single-vessel disease was attenuated after adjustment (adjusted odds ratio, 0.74; 95% CI, 0.23 to 2.34; P = 0.604). The adjusted model for angiographic thrombus presence was not estimable because only 7 patients lacked thrombus. Regression results are shown in Tables 5 and 6.

**Table 5:** Multivariable logistic regression for patient-level angiographic and procedural outcomes.

Outcome	Events in SUD group	Events in non-SUD group	Unadjusted OR (95% CI)	Unadjusted P	Adjusted OR (95% CI)	Adjusted P
Single-vessel coronary disease	50/75 (66.7)	38/100 (38.0)	3.26 (1.74 to 6.11)	P < 0.001	0.74 (0.23 to 2.34)	P = 0.604
Glycoprotein IIb/IIIa inhibitor use	33/75 (44.0)	24/100 (24.0)	2.49 (1.30 to 4.75)	P = 0.005	0.31 (0.09 to 1.06)	P = 0.062
Angiographic thrombus presence	72/75 (96.0)	96/100 (96.0)	1.00 (0.22 to 4.61)	P = 1.000	Not estimable	Not estimable

OR, odds ratio; CI, confidence interval; SUD, substance use disorder. Adjusted models included age, sex, current smoking, diabetes mellitus, hypertension, chronic kidney disease, admission systolic blood pressure, and chest-pain duration. The adjusted model for angiographic thrombus presence was not estimable because only 7 patients lacked angiographic thrombus.

**Table 6:** Multivariable linear regression for continuous echocardiographic outcomes.

Outcome	Unadjusted beta (95% CI)	Unadjusted P	Adjusted beta (95% CI)	Adjusted P
Left ventricular ejection fraction, percentage points	1.29 (− 0.90 to 3.48)	P = 0.249	− 0.44 (− 4.74 to 3.85)	P = 0.839
Number of affected cardiac segments	− 0.10 (− 0.72 to 0.52)	P = 0.757	0.97 (− 0.21 to 2.15)	P = 0.109

Beta coefficients represent the adjusted mean difference associated with substance use disorder compared with no substance use disorder. Linear models used heteroskedasticity-consistent standard errors. Covariates were the same as in Table 5.

Adjusted estimates were obtainable for both logistic models, but their stability was limited. Confidence intervals were wide, and the adjusted association for glycoprotein IIb/IIIa inhibitor use changed direction relative to the unadjusted estimate, consistent with strong confounding and sparse-data instability rather than evidence of a protective association. No internal or external validation was performed, and the models should not be used for individual prediction or definitive estimation of an independent substance use disorder effect.

## Discussion

This study adds to the literature on premature coronary artery disease by focusing on patients presenting with STEMI and undergoing primary PCI in a setting where cannabis, tramadol, opium, and polysubstance exposure are clinically relevant. The central observation is not that substance use disorder causes STEMI, which cannot be inferred from this observational design, but that patients with substance use disorder presented with a distinct unadjusted clinical profile. After multivariable adjustment, however, substance use disorder was not independently associated with the principal angiographic or procedural outcomes assessed. This attenuation is not a statistical inconvenience; it is the main interpretive safeguard of the revised manuscript.

The younger age of patients with substance use disorder is consistent with prior studies of substance exposure among patients with premature myocardial infarction^10–13,24,25^. The mean age difference between groups was 20.6 years, women were present only in the non-substance use disorder group, and current smoking was universal in the substance use disorder group. This degree of non-overlap is not a minor baseline imbalance. It limits practical positivity and prevents regression adjustment from reconstructing a fully comparable counterfactual group. The adjusted estimates therefore remain vulnerable to residual confounding and should not be interpreted as clean independent effects of substance use disorder.

The substance profile was dominated by cannabis and tramadol, followed by opium and polysubstance use. This pattern is relevant to the local clinical setting and differs from many cohorts dominated by cocaine or amphetamine exposure. Mechanistically, cannabis has been associated with sympathetic stimulation, altered vascular tone, platelet activation, and prothrombotic effects, whereas opioids and tramadol may influence autonomic tone, rhythm stability, drug absorption, and platelet biology during acute coronary syndromes^8,9,12,26^. These mechanisms are biologically plausible but cannot be proven by the present angiographic dataset.

Patients without substance use disorder had a significantly higher prevalence of atherosclerotic risk factors, including diabetes, hypertension, chronic kidney disease, and chronic liver disease, except for smoking, which was universal among patients with substance use disorder. These findings are consistent with Mousavi et al.^24^, Mansour et al.^12^, Genet et al.^27^, and Brgdar et al.^13^, who reported younger age and fewer conventional risk-related co-morbidities among patients with illicit drug or substance use exposure.

Admission blood pressure, dyspnea, fine basal crepitations, blood urea, creatinine, and mitral regurgitation were more prominent among patients without substance use disorder. A likely explanation is the older age and greater cardiometabolic and renal co-morbidity burden in the non-substance use disorder group. Prior hematological studies in the substance use disorder population described altered leukocyte and inflammatory indices, including an elevated neutrophil-to-lymphocyte ratio, although those studies were conducted among STEMI population^35–38^. These findings should not be interpreted as protective or causal effects of substance use disorder.

Anterior infarction was the most common STEMI territory in both groups. This finding is broadly consistent with studies of younger STEMI cohorts and substance-exposed cohorts reporting frequent anterior and anterolateral infarction^12,24^. Left ventricular systolic and diastolic function did not differ significantly between groups, consistent with prior reports showing broadly similar LVEF among patients with and without opium or other substance exposure in acute coronary settings^28–31^.

The angiographic pattern in the substance use disorder group, particularly more frequent single-vessel disease and greater glycoprotein IIb/IIIa inhibitor use in unadjusted comparisons, is compatible with a thrombus-focused procedural phenotype in younger patients with fewer chronic atherosclerotic co-morbidities. However, the adjusted models weakened this interpretation. The high thrombus frequency in both groups indicates that thrombosis is intrinsic to STEMI rather than unique to substance use disorder, and glycoprotein IIb/IIIa inhibitor use remains a treatment decision influenced by operator judgement, lesion features, bleeding risk, and procedural circumstances. It should not be interpreted as a direct angiographic thrombus burden score. Contemporary studies are similarly inconsistent: Amirpour et al. found no difference in post-PCI TIMI flow or in-hospital outcomes among opioid-dependent patients^14^, whereas Clement et al. reported higher adjusted in-hospital major adverse cardiac events and ventricular arrhythmias among patients with recent recreational drug exposure^15^. Longer-term PCI data have also associated opium consumption with increased mortality and major adverse cardiac and cerebrovascular events^17^. The inconsistency across studies supports substance-specific, prospectively adjudicated research rather than a single pooled causal interpretation.

Available rhythm-derived arrhythmia or conduction abnormality did not differ meaningfully between groups. Prior studies have reported ventricular arrhythmias among patients with opium dependence after myocardial infarction^32,33^, but the present dataset does not support a strong between-group rhythm difference. Renal injury markers were higher among patients without substance use disorder, consistent with their older age and greater chronic kidney disease burden. These laboratory markers should not be overinterpreted as adjudicated post-procedural acute kidney injury.

These findings suggest that patients with substance use disorder represent a clinically important subgroup of STEMI patients. Their lower burden of conventional co-morbidities should not be mistaken for the absence of procedural complexity. At the same time, the adjusted models argue against overclaiming an independent substance use disorder effect. The available in-hospital findings are hypothesis-generating and require confirmation in larger cohorts with standardized toxicology confirmation, formal thrombus scoring, intravascular imaging, adjudicated events, and long-term follow-up.

## Strengths and limitations

The strengths of this study include prospective consecutive screening, explicit exposure classification using DSM-5 criteria supported by urine toxicology screening, and a focus on a clinically underreported subgroup of STEMI patients undergoing primary PCI in a Middle Eastern setting.

This study has important limitations. First, it was a single-center observational study with a modest sample size, limiting statistical power and generalizability. The groups were markedly imbalanced in age, sex, smoking, diabetes, hypertension, chronic kidney disease, liver disease, and admission hemodynamics. All patients with substance use disorder were male and current smokers. This limited covariate overlap means that residual confounding is likely and that multivariable adjustment cannot identify a definitive independent substance use disorder effect.

Second, the adjusted models were exploratory and vulnerable to overfitting. The glycoprotein IIb/IIIa inhibitor model had only 57 events across 9 predictor terms, approximately 6.3 events per predictor, and sex, smoking, and chronic kidney disease showed complete or near-complete separation. No internal optimism correction, cross-validation, or external validation was performed. Wide confidence intervals, direction changes after adjustment, and failure of the thrombus model to estimate reliably indicate instability. The adjusted estimates should therefore be interpreted as sensitivity analyses, not as validated independent effects or prediction models. Rare in-hospital events were unsuitable for adjusted modeling and remain descriptive.

Third, 31 patients who died within 6 h of presentation were excluded because an accurate history and urine sample could not be obtained. This survivor-selection mechanism may have excluded patients with fulminant intoxication, cardiogenic shock, or malignant arrhythmia, thereby underestimating early severity and event rates. If substance use disorder was associated with early death, the exclusion could bias the exposure-outcome associations in an unknown direction. Exposure status was unavailable in these patients, so the magnitude of this bias cannot be assessed.

Fourth, substance use disorder classification relied on DSM-5 clinical criteria supported by a rapid qualitative urine immunoassay without confirmatory gas chromatography–mass spectrometry or liquid chromatography-mass spectrometry. Point-of-care assays have substance-specific detection windows, may show class cross-reactivity, cannot quantify dose or timing, may not distinguish therapeutic from nonmedical exposure, and do not detect substances outside the panel. Testing after admission, including up to 18 h after PCI, also creates potential false-negative or false-positive classification. Combining DSM-5 assessment with screening reduces reliance on either source alone, but exposure misclassification remains possible and could attenuate or distort associations.

Fifth, angiographic assessment did not include systematic SYNTAX scoring, formal thrombus grading, intravascular imaging, or core-laboratory adjudication. Several procedural and rare in-hospital outcomes were unavailable or insufficiently verified for robust comparative inference. Finally, follow-up was restricted to the early in-hospital period and did not evaluate long-term mortality, recurrent myocardial infarction, stent thrombosis, heart-failure hospitalization, abstinence, or substance-use treatment after discharge. These limitations require cautious interpretation and preclude causal inference.

## Conclusions

Patients with substance use disorder represented a substantial proportion of this STEMI cohort and were younger, exclusively male, and less burdened by traditional cardiometabolic co-morbidities than patients without substance use disorder. Coronary thrombosis was highly prevalent in both groups and did not distinguish the cohorts. Unadjusted differences in single-vessel disease and glycoprotein IIb/IIIa inhibitor use were attenuated after multivariable adjustment.

The association between substance use disorder and a thrombus-rich procedural phenotype should be interpreted as observational and hypothesis-generating rather than causal or independently proven. Larger multicenter studies with standardized toxicology confirmation, formal thrombus scoring, intravascular imaging, adjusted models, adjudicated in-hospital events, and long-term follow-up are needed.

## Data Availability

De-identified data are available from the corresponding author upon reasonable request and after approval by the relevant institutional authority.

## Abbreviations

ACS: Acute coronary syndrome
AF: Atrial fibrillation
AKI: Acute kidney injury
AMI: Acute myocardial infarction
CKD: Chronic kidney disease
DBP: Diastolic blood pressure
DES: Drug eluting stent
DM: Diabetes mellitus
DSM: Diagnostic and statistical manual of mental disorders
ESC: European society of cardiology
GIT: Gastro intestinal tract
HTN: Hypertension
LAD: Left anterior descending
LCX: Left circumflex
MR: Mitral regurgitation
PCI: Percutaneous coronary intervention
RCA: Right coronary artery
STEMI: ST-segment elevation myocardial infarction
SUD: Substance use disorders
SBP: Systolic blood pressure
TIMI: Thrombosis in myocardial infarction
VT: Ventricular tachycardia
